# Etramp5 as a useful serological marker in children to assess the immediate effects of mass drug campaigns for malaria

**DOI:** 10.1186/s12879-022-07616-8

**Published:** 2022-07-26

**Authors:** T. Druetz, L. van den Hoogen, G. Stresman, V. Joseph, K. E. S. Hamre, C. Fayette, F. Monestime, J. Presume, I. Romilus, G. Mondélus, T. Elismé, S. Cooper, D. Impoinvil, R. A. Ashton, E. Rogier, A. Existe, J. Boncy, M. A. Chang, J. F. Lemoine, C. Drakeley, T. P. Eisele

**Affiliations:** 1 Center for Applied Malaria Research and Evaluation, School of Public Health and Tropical Medicine, Tulane University, New Orleans, USA; 2 Department of Social and Preventive Medicine, School of Public Health, University of Montreal, Montreal, Canada; 3 Centre de Recherche en Santé Publique, Montreal, Canada; 4 Department of Infection Biology, London School of Hygiene and Tropical Medicine, London, UK; 5 Malaria Branch, Centers for Diseases Control and Prevention, Atlanta, USA; 6 CDC Foundation, Atlanta, USA; 7 IMA World Health, Port-au-Prince, Haiti; 8 Laboratoire National de Santé Publique, Port-au-Prince, Haiti; 9 Programme National de Contrôle du Paludisme, Port-au-Prince, Haiti

**Keywords:** Malaria, Serology, Seropositivity, Mass drug administration, Evaluation, Impact assessment, Cohort study, Haiti

## Abstract

**Introduction:**

Serological methods provide useful metrics to estimate age-specific period prevalence in settings of low malaria transmission; however, evidence on the use of seropositivity as an endpoint remains scarce in studies to evaluate combinations of malaria control measures, especially in children. This study aims to evaluate the immediate effects of a targeted mass drug administration campaign (tMDA) in Haiti by using serological markers.

**Methods:**

The tMDA was implemented in September–October 2018 using sulfadoxine-pyrimethamine and single low-dose primaquine. A natural quasi-experimental study was designed, using a pretest and posttest in a cohort of 754 randomly selected school children, among which 23% reported having received tMDA. Five antigens were selected as outcomes (MSP1-19, AMA-1, Etramp5 antigen 1, HSP40, and GLURP-R0). Posttest was conducted 2–6 weeks after the intervention.

**Results:**

At baseline, there was no statistical difference in seroprevalence between the groups of children that were or were not exposed during the posttest. A lower seroprevalence was observed for markers informative of recent exposure (Etramp5 antigen 1, HSP40, and GLURP-R0). Exposure to tMDA was significantly associated with a 50% reduction in the odds of seropositivity for Etramp5 antigen 1 and a 21% reduction in the odds of seropositivity for MSP119.

**Conclusion:**

Serological markers can be used to evaluate the effects of interventions against malaria on the risk of infection in settings of low transmission. Antibody responses against Etramp5 antigen 1 in Haitian children were reduced in the 2–6 weeks following a tMDA campaign, confirming its usefulness as a short-term marker in child populations.

**Supplementary Information:**

The online version contains supplementary material available at 10.1186/s12879-022-07616-8.

## Introduction

In areas of low malaria transmission, it is increasingly difficult to accurately measure the level of transmission due to the low number of parasite-positive individuals. Parasite prevalence, commonly used in endemic areas as an indicator of the burden attributable to malaria, becomes less accurate and therefore less meaningful as transmission decreases [1]. This problem is compounded by the fact that the rapid diagnostic tests (RDTs) commonly used in epidemiological surveys do not detect a significant proportion of subpatent infections: those with parasitemia densities so low that they are below the detection threshold [2].

New diagnostic tools have been developed to estimate malaria transmission in low-transmission or elimination settings without the need for surveys with a prohibitively large sample size. In these settings, serological markers have been increasingly used as indicators of a population’s exposure history, since antibody responses tend to last longer than infections in humans [3]. Although serosurveillance has been mostly used to assess cumulative exposure (by focusing on long-lasting antibodies), recent research has shown that antibody responses to some specific antigens were representative of current or recent exposure to *Plasmodium* [4, 5].

Serological methods provide metrics that have been proven useful in estimating not only age-specific period prevalence in low-transmission settings but also changes in malaria transmission [6, 7]. In rare instances, they have also been used to evaluate the effects of malaria control measures, including under trial conditions [8, 9]. However, evidence about the use and validity of seropositivity as an endpoint remains scarce in evaluation studies, especially to assess the immediate effects of an intervention. As more antigens inducing short-term antibodies are identified, this may become an increasingly feasible and useful tool for evaluating the impact of layered interventions and progress towards malaria elimination [10].

The aim of this study was to evaluate the immediate effects of a targeted mass drug administration campaign (tMDA) in Haiti by using serological markers. Using a retrospective cohort of school-aged children nested within a panel study, a quasi-experimental study (pre-post with nonrandomized control group) was established to: (1) assess the campaign’s effectiveness in reducing seropositivity to a set of *P. falciparum* antigens and (2) compare how different types of serological markers (short-term and long-term) were immediately affected by the tMDA campaign.

## Methods

### The tMDA campaign

With the assistance of the Malaria Zero Consortium, the National Malaria Control Program in Haiti has implemented a tMDA campaign using sulfadoxine-pyrimethamine (SP) and single low-dose (SLD) primaquine in a single round. The target dose for SP was 25/1.25 mg/kg, the approved therapeutic dose in Haiti for second-line treatment. The target dose for SLD primaquine was 0.25 mg/kg, lower than the recommended therapeutic dose and used in the tMDA to clear late-stage gametocytes. The campaign took place just before the peak of the annual rainy season, October 10–November 6, 2018, with the intention of accelerating progress towards malaria elimination [11, 12]. It was implemented in conjunction with an indoor residual spraying (IRS) campaign that used a pyrethroid insecticide (Actellic CS) [13].

The study was conducted in a pilot area covering five communes of the Grande-Anse Department (first-level administrative division), which is the Department (among 10) with the highest malaria incidence rate in the country (18.1 per 1000 in 2017) [14]. Within the pilot area, the tMDA campaign was delineated to 12 operational units, defined as the contiguous polygonal areas of approximately 2000 residents with the highest predicted reproductive numbers. Models that integrated population density, surveillance data, population mobility scores, and ecological factors were used to predict risk of transmission and rank operational units. The targeted areas that comprised these 12 operational units covered ~ 100 km^2^ with an estimated population of 46,372. All individuals in the targeted areas aged ≥ 6 months were offered directly observed, age-appropriate treatment of SP + SLD primaquine. Women in their first trimester of pregnancy and participants with signs of severe illness, known allergies to the treatment, specific medical conditions (e.g.: being in the first trimester of pregnancy), or using contraindicated medications (e.g.: sulfanomide or any other antimalarial medication) were excluded. More information about the tMDA/IRS campaign and the pilot area are available elsewhere [13, 15].

### Study design and recruitment

This study was conducted in 25 schools that serve as easy access groups; in comparison to population-based household surveys, logistical issues and costs are considerably reduced in easy access groups surveys, while they have proven to be effective proxies for measuring the effectiveness of malaria interventions [16]. The schools were selected by stratified random sampling after a census of all schools with at least 100 pupils in the pilot area (which includes but is not limited to the areas targeted for MDA). Equal distribution of the schools across communes and by remoteness was ensured. Surveys were conducted in 2017 (November 6–December 7, i.e., pretest) and 2018 (November 12–December 13, i.e., posttest). The only eligibility criterion was that the children had to be regularly enrolled in the school. All pupils present at school at the time of the survey were therefore eligible to participate, irrespective of their age or symptoms. The target sample size was 2500 participants each year, or ~ 100 children per school. When the total number of children present at a school exceeded 150, a simple random sample of 150 children were selected from the entire pupil population at that school. Only pupils present at the time of the survey were sampled; for logistical reasons, it was not feasible to return on another day to the site to survey children who were temporarily absent.

Since the same set of schools was surveyed both years, it was possible to identify a nested cohort of children. Matching between the 2017 and 2018 lists of participants was performed independently by two team members who compared the school, the children’s full name, and their date of birth. The present study is restricted to the matching subset of children who were surveyed twice. Among these, children who self-reported in the 2018 survey that they had taken the drugs distributed during the tMDA campaign constituted the exposure group; children who self-reported that they did not constituted the control group. This was a natural experiment conducted independently from the intervention; the study was not initially designed to assess the effects of the tMDA campaign.

More information about the recruitment procedures and the selection of the schools is available elsewhere [17]. Rate of refusal to participate in the survey was < 1% both years. The Strengthening the Reporting of Observational Studies in Epidemiology (STROBE) guidelines were followed.

### Survey procedures

A sociodemographic questionnaire was administered to all participating children, with standardized questions about their age, history of fever, use of bed nets, travel, treatment-seeking practices, and exposure to tMDA. Siblings from the same household were gathered together, and the oldest sibling was asked to help the younger ones with their responses. Data were automatically entered on a mobile data collection platform installed on tablets and uploaded to a secure cloud-based server.

A capillary blood sample from a finger prick was taken to perform a conventional histidine-rich protein 2 (HRP2)-based RDT (Standard Diagnostic Bioline Ag. Pf, South Korea) to test for *P. falciparum* parasite infection. Finger prick blood was also spotted on Whatman 903 cards (GE Healthcare), dried overnight at ambient temperature, and packed the next day with silica gel. Dried blood spots were stored at + 4 °C and transported to the national laboratory (*Laboratoire National de Santé Publique*) in Port-au-Prince weekly.

### Laboratory procedures

A set of 17 *P. falciparum* antigens were included in the panel, with antigens covalently coupled to unique bead regions [18]. Complete information regarding the creation and production of this panel of P. falciparum antigens has been published previously [4, 10, 18]. Immunoglobulin G data were collected for all participants. Antibody levels were measured using a bead-based assay in which sample and secondary antibody were incubated simultaneously overnight. Median fluorescence intensity (MFI) was recorded and corrected for background reactivity to give a final value of MFI minus background. Alongside participant samples, a six-point serial dilution of a hyperimmune positive control pool was added to each plate to assess plate-to-plate variation in data collection. More information about the laboratory procedures, including quality control, is available elsewhere [10, 18, 19].

### Serological outcomes

Antibody concentrations from the 2017 and 2018 surveys were aligned using a procedure detailed elsewhere [10]. Seropositivity was defined separately for each antigen using a mixture model approach. Two distributions were identified in the log-transformed median fluorescence intensity (MFI) data, and the threshold for positivity was set at the mean of the lower distribution plus five standard deviations [19]. Based on an antigen selection process described elsewhere [10], five antigens were selected for further analysis: MSP1-19, AMA-1, Etramp5 antigen 1 (ETR51), HSP40, and GLURP-R0. Among these, three (HSP40, GLURP-R0, and ETR51) were previously shown to be short-term markers, defined as informative of recent exposure (i.e., exposure that occurred within the previous three months) [5, 20]. ETR51 showed the highest accuracy in predicting current or recent infection to *P. falciparum* in the Haitian context [4, 5, 10]. The remaining two antigens (MSP1-19 and AMA-1) are usually considered as long-term markers in the adult population, but results are less conclusive in children and they were therefore added to the analysis [21–23].

### Analyses

Exposure to tMDA was assessed by each pupil’s self-report. Descriptive analysis to assess pre-post differences in socio-demographic characteristics between the intervention and comparison groups was performed using Chi-square tests or Fishers’ tests, for frequencies, and t-tests, for means. For each of the five antigens, a multilevel logistic regression model was fitted with seropositivity of the participants as the dependent variable. A set of time-varying potential confounding variables were tested in the models: socioeconomic characteristics of the households (size, occupation of the head of household, owning of livestock and bed nets), household’s exposure to indoor residual spraying in the previous three months, individual use of a bed net the night before, travel history, and total rainfall at the recruitment site during the previous two months. To facilitate comparison, the same set of variables was kept in the five final models, with the best-fitting model selected according to the Akaike information criterion values. Random intercepts at the individual, household, and commune levels were also included, and robust variance estimators were used across all analyses [24, 25].

Treatment effects were assessed following a difference-in-differences approach, which allows controlling for observed and unobserved time-invariant confounders [26]. Pre-post changes in seropositivity were therefore compared between the exposure and control groups by adding an interaction term between time period and exposure. Because impact estimates sometimes vary depending on the type of measure, three different indicators were used in the analysis as recommended: risk difference, odds ratio, and relative risks [27]. Relative risks were derived from logistic regression models by computing marginal standardized probabilities and using the margins and nlcom Stata commands [28]. All analyses were performed using Stata version 14.0 software (StataCorp LLC, College Station, Texas).

### Ethical considerations

The study was approved by the National Bioethics Committee in Haiti (1516–30), the London School of Hygiene and Tropical Medicine Ethics Committee (103939), and the Tulane University Institutional Review Board (795709). Participation in the study was not remunerated. Activity did not constitute engagement in human subjects research as determined by the human subjects office of the US Centers for Disease Control and Prevention Center for Global Health (2016-135a).

Written assent was sought for children > 6 years of age. Participants could choose to give thumbprint consent/assent if they could not sign. An opt-out method was used to obtain consent from the children’s parents, as described elsewhere [17]. Written informed consent was also obtained from each school director after consultations and consent from the Department of Education and local leaders.

## Results

### Study participants and tMDA exposure

A total of 872 children were successfully matched between the two surveys. About 66% (n = 580) of these children reported not having been exposed to tMDA, 20% (n = 174) reported having been exposed, and 13% (n = 118) were unsure and were removed from further analysis leaving 754 children for this study (for a total of 1508 year-observations, ~ 30% of the original enrolled cohort). Table 1 presents the time-variant and -invariant sociodemographic characteristics of the participants and their households, according to the year of the survey and the exposure group. Less than 5% (n = 9) of children living in households who were targeted and visited during the tMDA campaign did not take the drug. At baseline, only 4 (< 1%) and 7 (4%) children presented a positive RDT in the control and intervention group, respectively; no children had a positive RDT in 2018.

**Table 1:** Sociodemographic characteristics of the participants, by year and exposure group

	Control group		P value^a^	tMDA group		P value^a^
	2017	2018		2017	2018	
*Time-invariant*						
Number	580	580		174	174	
Female	0.45			0.52		
Commune						
Moron	0.25			0.01		
Chambellan	0.25			0.04		
Dame-Marie	0.41			0.18		
Anse-d’Hainault	0.08			0.70		
Les Irois	0.01			0.07		
*Time-variant*						
Age (mean)^b^	10.4	11.4	< 0.001	11.3	12.3	< 0.001
Slept under a bed net the night before	0.45	0.37	0.005	0.53	0.49	0.453
History of fever (last 2 weeks)	0.02	0.05	0.009	0.02	0.05	0.190
RDT positive	0.01	0.00	0.005	0.04	0.00	0.008
Occupation of the head of the household						
Farmer	0.83	0.74	< 0.001	0.78	0.76	0.064
Shop keeper	0.10	0.12		0.10	0.17	
Other	0.07	0.01		0.12	0.07	
Large household (> 5 members)	0.61	0.66	0.077	0.63	0.72	0.086
Household owns ≥ 1 bed net	0.53	0.53	0.076	0.62	0.70	0.142
Household owns livestock	0.62	0.73	< 0.001	0.49	0.66	0.002
Household received IRS (last 3 months)	0	0.10	< 0.001	0	0.58	< 0.001

*tMDA* targeted mass drug administration, *RDT* rapid diagnostic test, *IRS* indoor residual spraying

^a^Chi-2 test (or t-test for continuous variable) on within-group difference

^b^Age ranged 5–19 years old. Ranges were similar across groups

### Effects on antibody level concentration

**Fig. 1:**
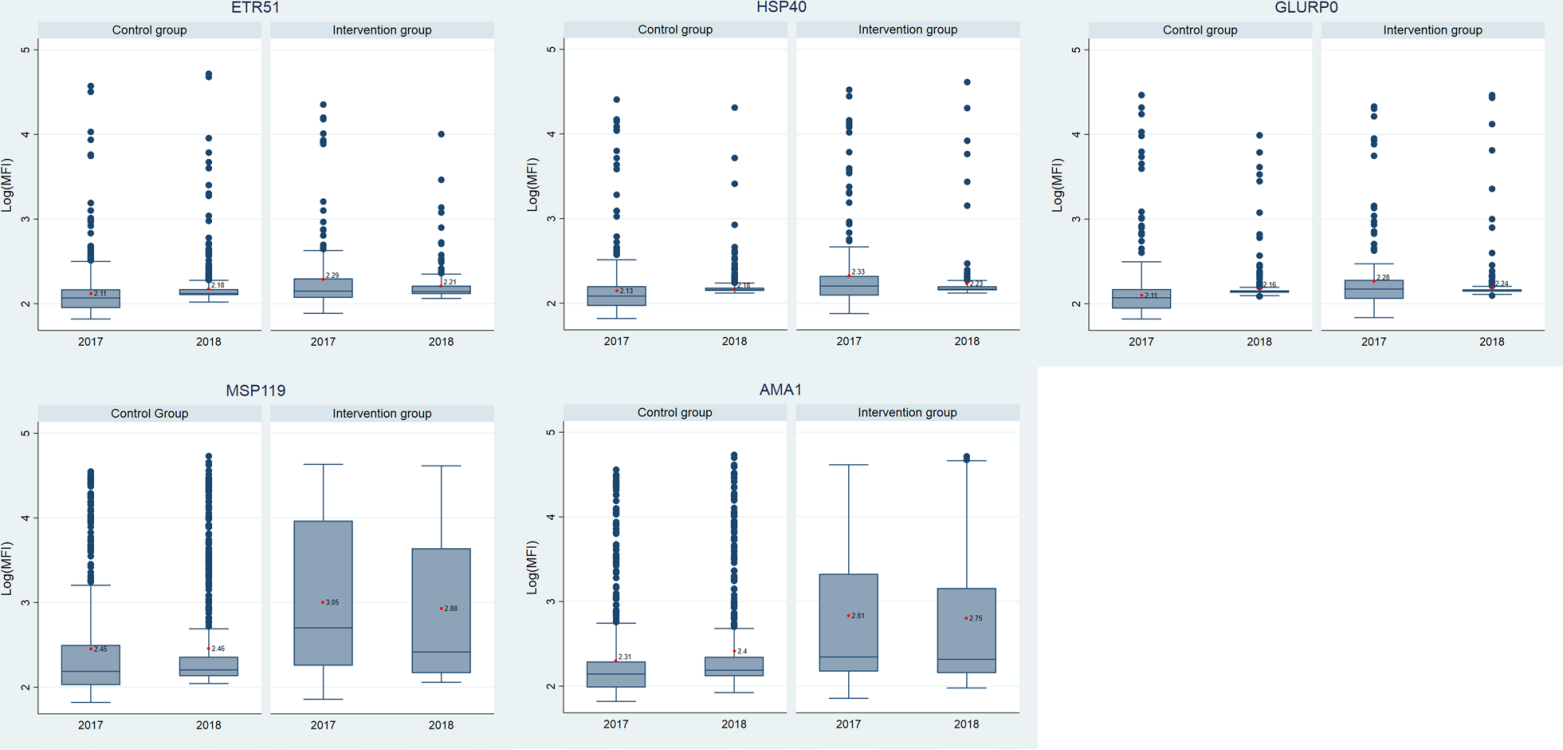
Box plots of normalized antibody concentration level for five *P. falciparum* antigens (2018 vs. 2017, by exposure group). Antibody concentration level is expressed by the median fluorescence intensity (MFI) after log-transformation and standardization between years for titre concentration. Mean concentration per year per group is displayed by red circles

Box plots show the differential distributions of antibody concentrations between the control and the exposure groups, and between the two periods (Fig. 1). For the five antigens under study, mean individual changes in antibody concentration levels between 2017 and 2018 were positive for the children belonging to the control group but negative for the children in the exposure group (Additional file 1). Using regression models with fixed effects at the individual level, the reductions attributable to the tMDA campaign were all statistically significant and ranged from − 0.08 log_10_(MFI) for GLURP-0 to − 0.18 log_10_(MFI) for MSP119 (Additional file 1).

Chi-square analyses suggest that the likelihood of a fourfold increase in individual antibody level concentrations between 2017 and 2018 was not different between the two groups. However, fourfold decreases in individual antibody level concentration were statistically significant in the exposure group compared to the control group for all the antigens but GLURP0 (Additional file 1).

### Effects on seropositivity

**Fig. 2:**
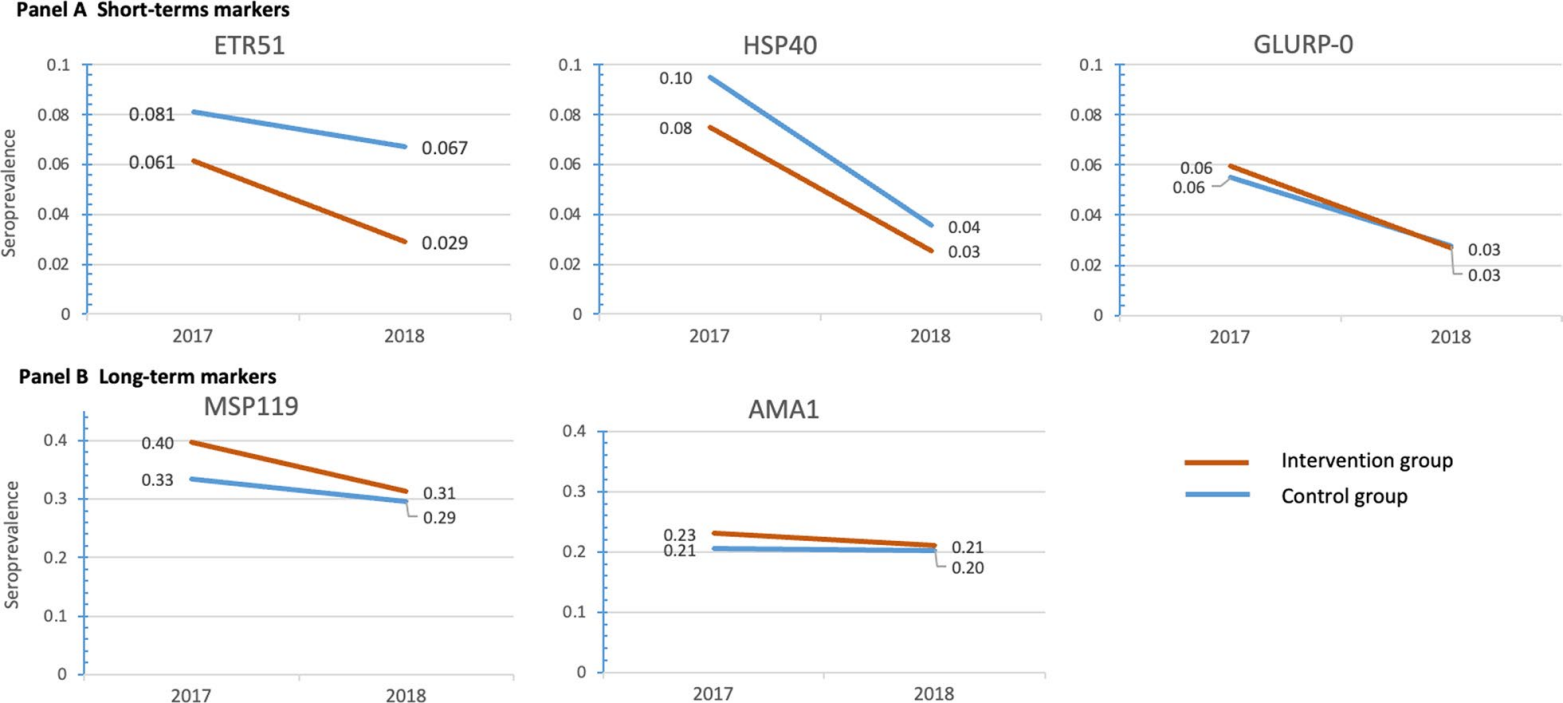
Changes in seroprevalence for five *P. falciparum* antigens (2018 vs 2017, by exposure group). Seroprevalence was estimated by fitting multilevel logistic regression models, with adjustment for age, use of a bednet the night before, size of the household, occupation of the head of the household and possession of livestock. Robust variance estimators were used, and models used random intercepts at the individual, household and commune levels. The intervention group refer to the individuals who self-reported having received MDA in 2018, while the control group refer to individuals who self-report not being exposed to MDA in 2018. No participant was exposed to MDA in 2017, whatever the group

**Fig. 3:**
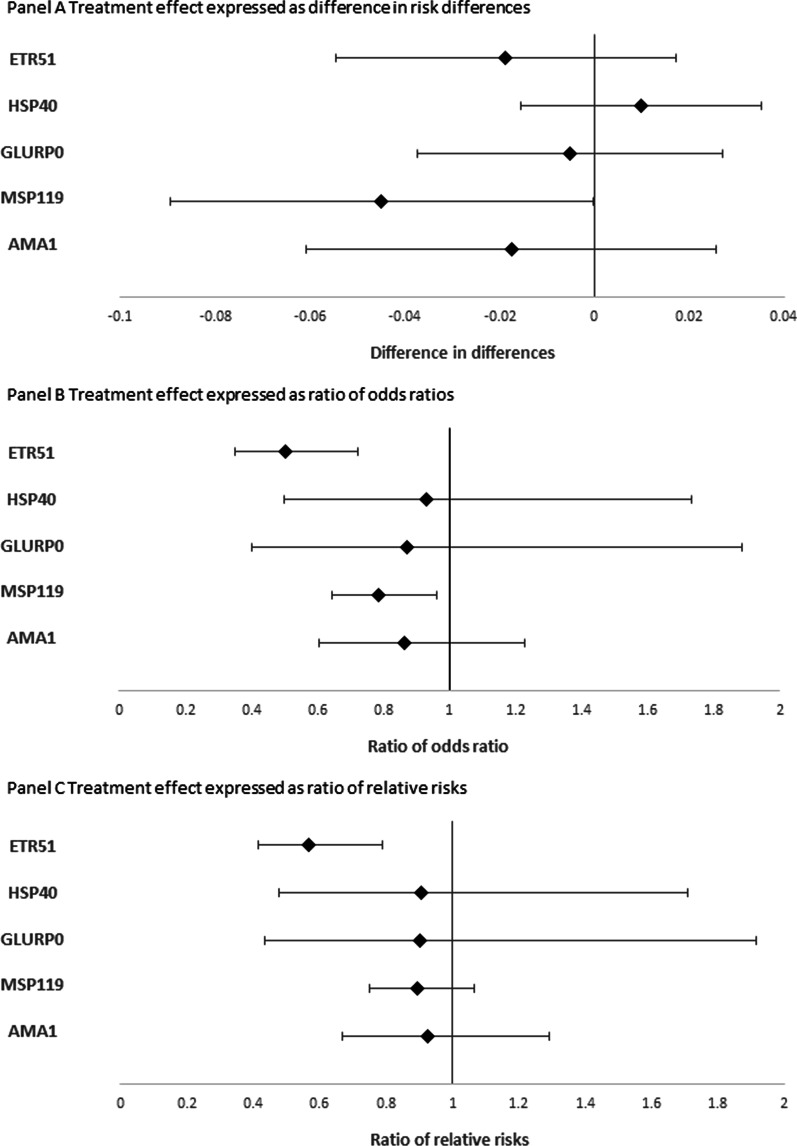
Treatment effects of MDA campaign on IgG seropositivity to five *P. falciparum* antigens. Treatment effects estimates are derived from multilevel logistic regression models, with adjustment for age, use of a bednet the night before, size of the household, occupation of the head of the household and possession of livestock. Robust variance estimators were used, and models used random intercepts at the individual, household and commune levels. Marginal probabilities were used for computing risk differences and relative risks. Treatment effect estimates are displayed with their 95% confidence intervals

Estimated seroprevalence was < 10% for the three antigens pre-identified as short-term: ETR51, HSP40, and GLURP-0 (Fig. 2, panel A). As expected, seroprevalence was higher for the longer-term markers AMA1 and MSP119, ranging from 20–23% and 29–40% according to the year and exposure group, respectively (Fig. 2, panel B). Pre-MDA, there was no statistical difference in seroprevalence between exposure and control groups for all antigens. Estimated seroprevalence decreased in all groups from the pre- to post-test. However, in absolute terms, the pre-post changes were not statistically different between the exposure and control groups, with the exception of antigen MSP119, for which seroprevalence was further reduced in the exposure group by 4.5% [95% CI 0.001; 0.090] (Fig. 3, panel A).

Treatment effects were also expressed as the ratio of adjusted odds ratios (RaOR) (Fig. 3, panel B). The model confirms that the tMDA campaign significantly decreased the odds of seropositivity to MSP119 (RaOR: 0.79, 95% CI [0.643; 0.960]), but also to ETR51 (RaOR: 0.50, 95% CI [0.349; 0.722]). Ratio of relative risks are available for comparison purposes (Fig. 3, panel C); only the reduction in seropositivity to ETR51 remains statistically significant (RRR: 0.567, 95% CI [0.444; 0.730]).

## Discussion

Using serological markers collected from a cohort of children before and after tMDA, we find that some *P. falciparum* serological markers can be informative of recent changes in individual risk of infection in children, as rapidly as 2–6 weeks after an intervention. This approach does not pretend to estimate an intervention’s impact on malaria prevalence; however, considering power issues and other difficulties to obtain precise estimates of point prevalence (current infection) in settings with low transmission, serological indicators can be used as a proxy for period prevalence (recent infection) [29, 30]. In the present study, the effects of the tMDA campaign could not have been interpreted using RDT results as endpoints, due to paucity of positive cases at pre- or post-test. An advantage of serological markers is their increased sensitivity, since antibody responses can be detected even for infections with parasitemia density so low that they would likely be missed by RDTs [31, 32].

The results suggest that, in Haitian children, antibody responses to ETR51 were rapidly and significantly affected by the tMDA campaign. The reduction in the odds of seropositivity was two times larger in exposed vs. non-exposed children, which corresponded to a 50% reduction in risk of recent infection attributable to the intervention. This estimate is lower than the 86–97% reduction in risk of current infection that previous studies have observed within one month post-MDA [15, 33]. This is consistent with the fact that the immediate effects of an MDA campaign would be larger on point prevalence than on period prevalence, because in the absence of a reinfection, some children would need more than a few weeks to revert to seronegative after parasite clearance from the bloodstream. Our results also corroborate previous studies that have identified ETR51 as representative of current or recent exposure to malaria parasite infection, especially in children [10, 32].

Surprisingly, the effects measured on the other two markers of recent infection, HSP40 and GLURP-0, were modest and not statistically significant. Pre-MDA, seroprevalence against them was comparable to seroprevalence against ETR51, which suggests that they are informative of recent infection. One hypothesis is that infections to *P. falciparum* induce antibody responses more rapidly for ETR51 than for HSP40 and GLURP-0; post-MDA, recent infections in the control group would not have had enough time to affect the latter two.

Interestingly, the tMDA campaign significantly affected the MSP1-19 antigen, which is usually considered a marker of long-term infection. The effects were more moderate (RaOR of ~ 0.8) and arguably reached statistical significance mainly because of the higher MSP1-19 seroprevalence at baseline. Another hypothesis is that MSP1-19 can be an indicator of recent infection in children, as has been suggested in other settings of low malaria transmission [21–23]. This would be consistent with the fact that antibody responses (including but not limited to MSP1-19) usually have a shorter half-life in children than in adults, as several boosts are required to maintain levels at which individuals would remain seropositive [3]. However, the higher seroprevalence against MSP1-19 in this cohort of children suggests that it was representative of a longer duration of exposure compared to the other short-term antigens.

The reductions in seropositivity are likely a combined effect of the preventive and therapeutic properties of the treatment administered during the tMDA campaign, although it is impossible to formally distinguish them in the present study design. The fact that both short-term and long-term antibody levels were reduced by the campaign suggests a decrease of recent seroconversion in the exposure group compared to expected. In contrast, the larger and more significant reduction in seropositivity for the short-term marker ETR51 indicates that a drop in antibody levels following parasite clearance is plausible, despite the short lapse of time between the campaign (Oct. 10–Nov. 6, 2018) and the survey (Nov. 12–Dec. 13, 2018).

Results suggest an overall reduction in seroprevalence between 2017 and 2018, both in the intervention and control groups. This result is consistent with the nearly three-fold reduction observed between 2017 and 2018 in the number of confirmed cases reported by the health facilities in Grande-Anse (from 8627 to 2937). This reduction was likely caused by several interrelated factors, among which the decrease in the total annual precipitation recorded in the department (from 2017 mm in 2017 to 1438 mm in 2018). Rainfall and forest cover have shown to be associated to risk of malaria infection in the area, although the evidence about the influence of environmental factors (including hazards) on malaria transmission in Haiti remains scarce and inconclusive [14, 34]. The gradual deployment of community health workers in Grande-Anse and sustained efforts to make healthcare free of charge for malaria patients may also have improved access to treatment and contributed to reduce transmission. In the end, it is difficult to exactly know the contributory factors because of the considerable spatio-temporal heterogeneity in Haiti, both for rainfall and malaria transmission [34, 35].

Three different measures were presented to assess the effects of the tMDA campaign based on odds ratios, risk differences, and risk ratios between the two groups. There is no gold standard between them when analyzing binary data: a particular measure is often selected based on scientific or statistical rationale (notably related to the type of study design and the objective), but it can also reflect preferences depending on interpretability or comparability considerations [36]. Due to a plateau effect, estimators based on absolute variations, such as the difference in risk differences, will arguably be less useful for measuring the impact of an intervention as seroprevalence decreases [37]. We therefore dispute the argument that the absolute scale is the most appropriate scale for inferring public health implications: Elimination programs are derived from a public health perspective [38]. Unsurprisingly, in this study, the largest (and the only statistically significant) effect as expressed by the difference in risk differences concerns the MSP1-19 antigen, for which seroprevalence was the highest at baseline in both groups.

Regarding the estimators based on a ratio scale, the ROR and RRR provided similar information for most of the antigens, especially those with low seroprevalence; this is understandable given that odds ratio and relative risks converge as the event under study becomes less likely [39]. When compared with the ROR, the RRR has the advantage of being interpreted as a change in individual risk likelihood, but requires transforming the estimator using marginal predicted probabilities, which can lead to some issues depending on the type of prediction used [28].

### Strengths and limitations

Several steps were taken to increase the internal validity of this evaluation. First, a cohort of children was identified within a panel study of schools, allowing for a robust evaluation design (a pre-post with a control group) [40]. The hierarchical structure of the data was taken into account, with random intercepts at the individual (observations nested within children), household, and commune levels. Time-invariant observable and non-observable confounding factors were controlled for by following a difference-in-differences approach, and the influence of potential time-variant confounding variables was tested in the models [26, 41]. In addition, three different measures of effects were assessed, following recent recommendations that arose from the fact that impact estimates can significantly vary from one measure to another [42]. Analyses were conservative: Bilateral tests were used throughout the study, and models were fitted with robust variance estimators [24]. Seropositivity was defined using conservative thresholds. Other interventions were implemented between the two surveys, and we cannot exclude the possibility that they affected our estimates; however, the context was closely monitored, and exposure to the main other types of interventions (IRS and bed net distribution) was controlled for in the models. In addition, if present, this potential bias would unlikely be differential according to the exposure status to tMDA.

It was not possible to test the assumption that trends were similar between the exposure and control groups during the pre-baseline period, nor to run fixed effects models for seropositivity at the individual level (due to the small number of pre-post serodiscordant pairs) [43]. However, it is reassuring that seroprevalence estimates for each of the five outcomes were not statistically different at baseline between the two groups. Since participants were school-aged children, some information bias might have occurred despite the measures taken to minimize this risk [15, 17]. However, nothing indicates that this bias, if present, would be differential according to the seropositivity status of the child or the tMDA exposure status. Any bias would likely be non-differential and thereby direct the results toward the null effect. Unfortunately, the natural experimental design made it impossible during the posttest to accurately measure the time elapsed (in days) since exposure to MDA. Finally, while a violation of the stable unit treatment value assumption (i.e., spillover effects) cannot be excluded, it is unlikely due to the short lapse of time between the intervention and the survey [42]. Further research with closer (i.e., daily) follow-up of participants in a cohort study should be conducted to determine the earliest time after an intervention when immunological markers are affected.

## Conclusion

Serological markers can potentially be used to evaluate the effects of interventions against malaria parasite infections in settings of low transmission. In the present cohort of Haitian children, antibody responses against ETR51 were rapidly affected by a tMDA campaign using SP + SLD primaquine. Seropositivity to ETR51 was significantly reduced in the 2–6 weeks following the intervention, confirming its usefulness as a short-term marker in child populations.

## Supplementary Information

**Additional file 1.** Changes in individuals’ normalized antibody concentration level for five *P. falciparum* antigens (2018 vs. 2017, by exposure group). Antibody concentration level is expressed by the median fluorescence intensity (MFI) after log-transformation and standardization between years for titre concentration. Mean individual changes between 2017 (pre-MDA) and 2018 (post-MDA) are displayed by the linear regression coefficients.

**Additional file 2.** Effects of tMDA on antibody concentration levels using logistic regression models with fixed effects at the individual level.

**Additional file 3.** Bivariate association between exposure to tMDA in 2018 and fourfold increases (or fourfold decreases) in MFI in 2018 compared to 2017.

## Data Availability

All anonymized data and Stata scripts used for this analysis can be made available by contacting the corresponding author under reasonable request.

## Abbreviations

CI: Confidence interval
DRD: Difference in risk differences
MFI: Median fluorescence intensity
OR: Odds ratio
PCR: Polymerase chain reaction
RaOR: Ratio of adjusted odds ratio
RDT: Rapid diagnostic test
RRR: Ratio of relative risks
tMDA: Targeted mass drug administration
AMA1: Apical membrane antigen 1
ETR51: Early transcribed membrane protein (Etramp5 antigen 1)
GLURP0: Glutamate rich protein R0
HSP40: Heat shock protein 40
MSP119: 19KDa fragment of MSP1 molecule
