# Supplementary material for: Etramp5 as a useful serological marker in children to assess the immediate effects of mass drug campaigns for malaria
Source: BMC Infect Dis. 2022 Jul 26;22:643. doi: 10.1186/s12879-022-07616-8 (PMC9321307; doi:10.1186/s12879-022-07616-8)
Supplement: Supplementary file 1 — Additional file 1. Changes in individuals’ normalized antibody concentration level for five P. falciparum antigens (2018 vs. 2017, by exposure group). Antibody concentration level is expressed by the median fluorescence intensity (MFI) after log-transformation and standardization between years for titre concentration. Mean individual changes between 2017 (pre-MDA) and 2018 (post-MDA) are displayed by the linear regression coefficients. [file 12879_2022_7616_MOESM1_ESM.docx]

**Additional file 1 Changes in individual’s normalized antibody concentration level for five *P. falciparum* antigens (2018 vs. 2017, by exposure group)**


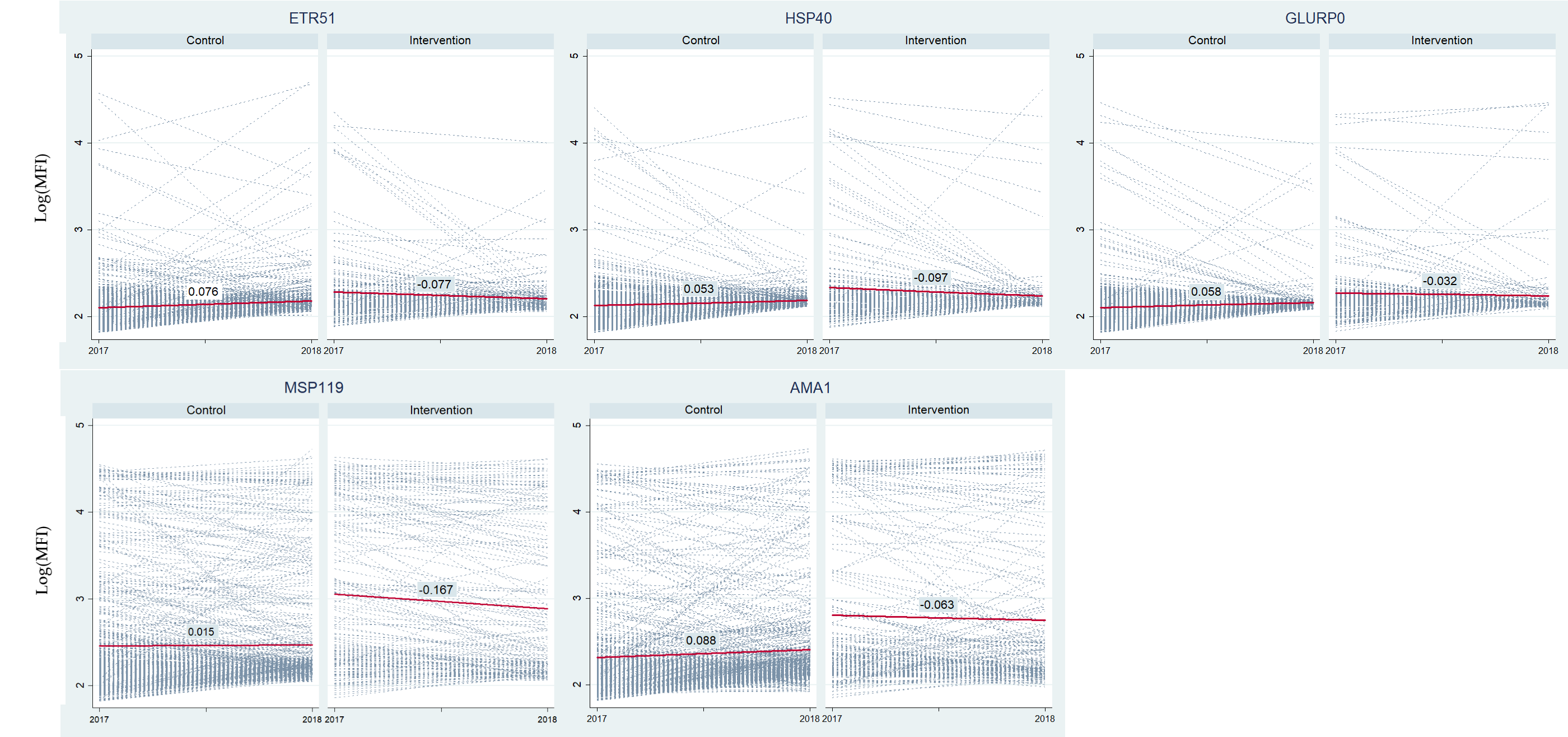


Antibody concentration level is expressed by the median fluorescence intensity (MFI) after log-transformation and standardization between years for titre concentrations. Mean individual changes between 2017 (pre-MDA) and 2018 (post-MDA) are displayed by the linear regression coefficients.
