# Supplementary material for: Etramp5 as a useful serological marker in children to assess the immediate effects of mass drug campaigns for malaria
Source: BMC Infect Dis. 2022 Jul 26;22:643. doi: 10.1186/s12879-022-07616-8 (PMC9321307; doi:10.1186/s12879-022-07616-8)
Supplement: Supplementary file 2 — Additional file 2. Effects of tMDA on antibody concentration levels using logistic regression models with fixed effects at the individual level. [file 12879_2022_7616_MOESM2_ESM.docx]

**Additional file 2 Effects of tMDA on antibody concentration levels using linear regression models with fixed effects at the individual level**

| Outcome | Linear coefficient expressing the effects of tMDA | 95% CI |
| --- | --- | --- |
| ETR51 | -0.153 | -0.218 ⎯ -0.087 |
| HSP40 | -0.151 | -0.218 ⎯ -0.084 |
| GLURP0 | -0.090 | -0.148 ⎯ -0.032 |
| MSP119 | -0.182 | -0.253 ⎯ -0.111 |
| AMA1 | -0.152 | -0.236 ⎯ -0.068 |
