# Supplementary material for: Etramp5 as a useful serological marker in children to assess the immediate effects of mass drug campaigns for malaria
Source: BMC Infect Dis. 2022 Jul 26;22:643. doi: 10.1186/s12879-022-07616-8 (PMC9321307; doi:10.1186/s12879-022-07616-8)
Supplement: Supplementary file 3 — Additional file 3. Bivariate association between exposure to tMDA in 2018 and fourfold increases (or fourfold decreases) in MFI in 2018 compared to 2017. [file 12879_2022_7616_MOESM3_ESM.docx]

**Additional file 3**

Bivariate association between exposure to tMDA in 2018 and 4-times increases in MFI in 2018 compared to 2017

| Four-fold increase in MFI (Etr51) between 2017 and 2018 | No | Yes | Total |
| --- | --- | --- | --- |
|  |  |  |  |
| No | 563 | 172 | 735 |
| Yes | 10 | 2 | 12 |
|  |  |  |  |
| Total | 573 | 174 | 747 |

Pearson chi2 = 0.2997 P-value = 0.584

*****************************************************************

| Four-fold increase in MFI (HSP40) between 2017 and 2018 | No | Yes | Total |
| --- | --- | --- | --- |
|  |  |  |  |
| No | 572 | 173 | 745 |
| Yes | 1 | 1 | 2 |
|  |  |  |  |
| Total | 573 | 174 | 747 |

Pearson chi2 = 0.8005 P-value = 0.371

*****************************************************************

| Four-fold increase in MFI (GLURP0) between 2017 and 2018 | No | Yes | Total |
| --- | --- | --- | --- |
|  |  |  |  |
| No | 570 | 172 | 742 |
| Yes | 3 | 2 | 5 |
|  |  |  |  |
| Total | 573 | 174 | 747 |

Pearson chi2 = 0.7863 P-value = 0.375

*****************************************************************

| Four-fold increase in MFI (MSP119) between 2017 and 2018 | No | Yes | Total |
| --- | --- | --- | --- |
|  |  |  |  |
| No | 555 | 170 | 725 |
| Yes | 18 | 3 | 21 |
|  |  |  |  |
| Total | 573 | 174 | 747 |

Pearson chi2 = 0.9619 P-value = 0.327

*****************************************************************

| Four-fold increase in MFI (AMA1) between 2017 and 2018 | No | Yes | Total |
| --- | --- | --- | --- |
|  |  |  |  |
| No | 544 | 160 | 704 |
| Yes | 30 | 14 | 44 |
|  |  |  |  |
| Total | 573 | 174 | 747 |

Pearson chi2 = 1.9173 P-value = 0.166

Bivariate association between exposure to tMDA in 2018 and 4-times decreases in MFI in 2018 compared to 2017

| Four-fold decrease in MFI (Etr51) between 2017 and 2018 | No | Yes | Total |
| --- | --- | --- | --- |
|  |  |  |  |
| No | 565 | 163 | 728 |
| Yes | 8 | 11 | 19 |
|  |  |  |  |
| Total | 573 | 174 | 747 |

Pearson chi2 = 13.0639 P-value = 0.000

*****************************************************************

| Four-fold decrease in MFI (HSP40) between 2017 and 2018 | No | Yes | Total |
| --- | --- | --- | --- |
|  |  |  |  |
| No | 560 | 160 | 720 |
| Yes | 13 | 14 | 27 |
|  |  |  |  |
| Total | 573 | 174 | 747 |

Pearson chi2 = 12.7869 P-value = 0.000

*****************************************************************

| Four-fold decrease in MFI (GLURP0) between 2017 and 2018 | No | Yes | Total |
| --- | --- | --- | --- |
|  |  |  |  |
| No | 557 | 164 | 721 |
| Yes | 16 | 10 | 26 |
|  |  |  |  |
| Total | 573 | 174 | 747 |

Pearson chi2 = 3.4688 P-value = 0.063

*****************************************************************

| Four-fold decrease in MFI (MSP119) between 2017 and 2018 | No | Yes | Total |
| --- | --- | --- | --- |
|  |  |  |  |
| No | 544 | 156 | 700 |
| Yes | 29 | 17 | 46 |
|  |  |  |  |
| Total | 573 | 174 | 747 |

Pearson chi2 = 5.2156 P-value = 0.022

*****************************************************************

| Four-fold decrease in MFI (AMA1) between 2017 and 2018 | No | Yes | Total |
| --- | --- | --- | --- |
|  |  |  |  |
| No | 551 | 159 | 710 |
| Yes | 23 | 15 | 38 |
|  |  |  |  |
| Total | 573 | 174 | 747 |

Pearson chi2 = 5.8942 P-value = 0.015
